# CD9 Upregulation-Decreased CCL21 Secretion in Mesenchymal Stem Cells Reduces Cancer Cell Migration

**DOI:** 10.3390/ijms22041738

**Published:** 2021-02-09

**Authors:** Chia-Chu Hsieh, Szu-Chun Hsu, Ming Yao, Dong-Ming Huang

**Affiliations:** 1 Institute of Biomedical Engineering and Nanomedicine, National Health Research Institutes, Miaoli 35053, Taiwan; chiachu@nhri.edu.tw; 2 Department of Laboratory Medicine, National Taiwan University Hospital and College of Medicine, National Taiwan University, Taipei 100225, Taiwan; ssuchun@gmail.com; 3 Department of Internal Medicine, National Taiwan University Hospital and College of Medicine, National Taiwan University, Taipei 100225, Taiwan; yaomingmd@gmail.com

**Keywords:** cancer cell migration, mesenchymal stem cells, CD9, CCL21 chemokine, ionomycin, nanoparticles

## Abstract

Tetraspanin CD9 is widely expressed on various cell types, such as cancer cells and mesenchymal stem cells (MSCs), and/or cell-released exosomes. It has been reported that exosomal CD9 plays an important role in intercellular communications involved in cancer cell migration and metastasis. However, reports on the effect of the CD9 of MSCs or MSC-derived exosomes on cancer cell migration are still lacking. In this study, using a transwell migration assay, we found that both dextran-coated iron oxide nanoparticles (dex-IO NPs) and ionomycin stimulated exosomal CD9 expression in human MSCs (hMSCs); however, hMSCs could not deliver them to melanoma cells to affect cell migration. Interestingly, a reduced migration of melanoma cell line was observed when the ionomycin-incubated hMSC-conditioned media but not dex-IO NP-labeled hMSC-conditioned media were in the bottom chamber. In addition, we found that dex-IO NPs decreased cellular CD9 expression in hMSCs but ionomycin increased this. Simultaneously, we found that ionomycin suppressed the expression and secretion of the chemokine CCL21 in hMSCs. The silencing of CD9 demonstrated an inhibitory role of cellular CD9 in CCL21 expression in hMSCs, suggesting that ionomycin could upregulate cellular CD9 to decrease CCL21 expression and secretion of hMSCs, which would reduce the migration of B16F10, A549 and U87MG cancer cell lines due to chemoattraction reduction of CCL21. The present study not only highlights the important role of bone marrow-derived hMSCs’ CD9-mediated CCL21 regulation in cancer bone metastasis but also suggests a new distinct pharmaceutical strategy for prevention or/and therapy of cancer metastasis.

## 1. Introduction

Mesenchymal stem cells (MSCs), or multipotent mesenchymal stromal cells, have the potential of self-renewal and multilineage differentiation, as well as other advantages for clinical applications; they are among the mostly used cell type for regenerative medicine [1,2,3]. MSCs can migrate to injured sites in response to disease signals to benefit tissue regeneration through MSCs’ differentiation potential or the release of paracrine factors with pleiotropic effects [3,4,5]. MSCs also possess the capacity to migrate to tumor sites. Although MSCs have shown tremendous therapeutic potential in regenerative medicine, their therapeutic effect on cancer remains controversial [3,5,6,7]. In some studies, MSCs have been shown to promote tumor development, but in others they exerted an inhibitory effect through multiple mechanisms [6,7]. Similarly, as in regenerative medicine, MSCs exert their dual effects via direct cell–cell interaction and indirect paracrine mechanisms [3]. Recently it was proposed that MSCs mediate their therapeutic functions and pro- or anti-tumor activity in a paracrine rather than a cellular manner [5,8].

MSCs’ paracrine activity is one of main mechanisms contributing indirectly to their cancer regulation as well as to their therapeutic potential in regenerative medicine. MSCs have been shown to secrete multiple factors with pro- and anti-tumor effects, which can affect survival, proliferation, angiogenesis, immunoregulation and migration/metastasis via controlling a number of cellular pathways. These paracrine factors can be as soluble agents directly secreted or packaged in released nano-sized extracellular vesicles (EVs) into the extracellular space. Similar to MSCs, MSC-derived EVs can exert both pro- and anti-tumor effects. Exosomes have been explored as the best-defined player of MSC-derived EVs for mediating tumor development [3,5,8]. For instance, some studies have indicated that using engineered MSCs to secrete exosomes enriched with miRNAs (miR-146b and miR-143) could be an effective strategy for tumor cell migration inhibition [9,10]. In addition, MSC-derived EVs exert beneficial effects on a number of tissue-injury-related diseases [11]. Moreover, MSCs can mediate antiapoptotic, anti-inflammatory, antioxidative, antifibrotic, and antibacterial effects via paracrine activity [12,13].

Growing evidence suggests that MSC-derived exosomes can perform as paracrine mediators by transferring signaling molecules to recipient cells and exert several opposite effects on the development of various tumors [5,8]. Cancer cell migration is one of the critical steps of metastasis, which is the major cause of cancer morbidity and mortality. There are also controversial reports on whether MSC-derived exosomes suppress or promote tumor cell migration. Exosomes isolated from MSCs promoted Wnt signaling activation to facilitate the migration and proliferation of the breast cancer cell line MCF-7 [14]. Exosomes derived from gastric cancer MSCs were reported to transfer miR-221 to HGC27 cells, resulting in facilitating the proliferation and migration of these cells [15]. On the other hand, using modified MSCs, synthetic miR-124 and miR-145 mimics could be packaged into exosomes and delivered to glioma cells by exosomes via gap junction-dependent and contact-independent processes, which decreased the migration of glioma cells and the self-renewal of glioma stem cells [16].

Tetraspanin CD9 is widely expressed on the cell surface of various cell types and/or cell-derived exosomes. Controversially, the expression of CD9 in cancer cells has been reported to exert pro- and anti-migratory functions in cell migration, likely due to its modulatory activity toward associated integrin complexes and other transmembrane proteins [17,18,19]. Besides RNA cargoes as described above, exosomes can modulate many of the biological processes by delivering their protein cargoes to the recipient cells. For example, Miki et al have reported that CD9-postive exosomes from cancer-associated fibroblasts (CAF) could be taken into scirrhous-type gastric cancer cells and might stimulate the migration ability of these cancer cells, in which CD9 might also play a role on the uptake of CAF-derived exosomes by cancer cells [20]. CD9 proteins are also expressed on the surface of MSCs and MSC-derived exosomes; however, to our knowledge, there has been no report about the effect of MSCs’ exosomal CD9 on cancer cell migration.

**Figure 1:**
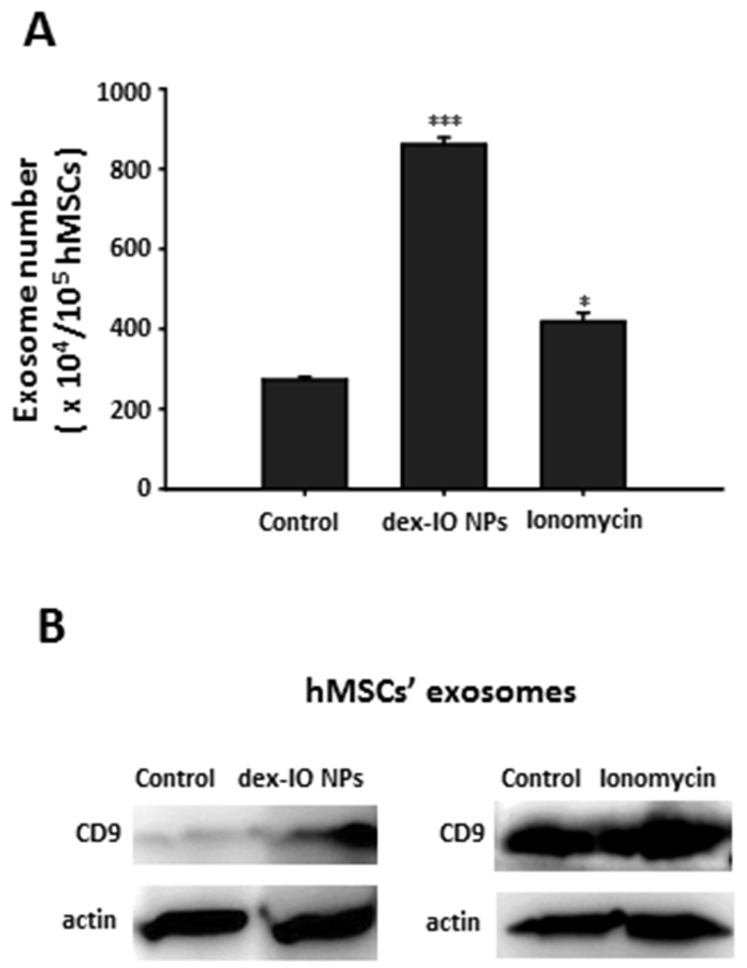
Effects of dex-IO NPs and ionomycin on hMSC’s exosome production and exosomal CD9 expression. (**A**) 1 × 10^5^ hMSCs were treated without (Control) or with dex-IO NPs for 1 h or ionomycin for 30 min followed by wash, and then exosomes released from hMSCs for 24 h were collected and determined by nanoparticle tracking analysis (NTA). Data are presented as the mean ± SEM of three independent experiments. * *p* < 0.05; *** *p* < 0.001 as compared with Control. (**B**) After treatment of hMSCs without (Control) or with dex-IO NPs for 1 h or ionomycin for 30 min, followed by wash, the exosomes released from hMSCs for 24 h were collected and analyzed by Western blot for exosomal marker CD9. Actin was used as a loading control. Data are representative of at least three independent experiments with similar results.

Previously our group has shown that the labeling of carboxydextran-coated superparamagnetic iron oxide (IO) nanoparticles (NPs), ferucarbotran (Resovist) used for in vivo magnetic resonance imaging (MRI) could promote the tumor tropism of human MSCs (hMSCs) through the induction of EGF receptor (EGFR) expression [21]. In addition, our previous data demonstrated that ferucarbotran was internalized into EGFR-localized endosomes and affected endosomal recycling, which could switch the intracellular traffic of EGFR from late endosomes to recycling endosomes, protect lysosomal degradation of EGFR, and result in exosomal EGFR overexpression [22]. Moreover, we synthesized dextran-coated IO NPs (dex-IO NPs) and found that dex-IO NPs could promote the tumor tropism of hMSCs in a B16F10 cancer cell model [23]. With regard to engineering MSCs to alter the impacts of exosomes on tumor migration [9,10,16], on the basis of our previous results we wondered whether dex-IO NPs could also alter MSCs’ exosomal cargoes to affect the impact of MSC-derived exosomes on cancer cell migration. Interestingly, we have found an upregulated expression of CD9 on dex-IO NP-labeled hMSC-derived exosomes (Figure 1). Since B16F10, A549, and U87MG cells are well used for cancer migration/metastasis investigation [24,25,26], in the study we explored the role of CD9 of hMSCs or hMSC-derived exosomes on the cancer cell migration of these three cell lines.

## 2. Results and Discussion

### 2.1. Dex-IO NPs and Ionomycin Stimulated hMSCs’ Exocytosis and Exosomal CD9 Expression

Because our previous study suggests that ferucarbotran could stimulate hMSCs’ exocytosis [22], we first demonstrated the stimulatory capacity of dex-IO NPs for exocytosis, which is indicative of an increasing number of exosomes (Figure 1A), and showed that dex-IO NPs could upregulate the expression of CD9 on hMSC-derived exosomes (Figure 1B). As previously reported, ionomycin, a calcium ionophore with the capacity for upregulated exocytosis in MSCs [27], was confirmed to be able to dramatically stimulate hMSCs’ exocytosis (Figure 1A) and to highly increase the CD9 expression on hMSC-derived exosomes (Figure 1B) and hence used to verify the possibility that upregulated CD9 on hMSC-derived exosomes can affect melanoma B16F10 cell [23] migration. Because neither dex-IO NPs nor ionomycin could increase hMSCs’ viability (Figure S1), the capacities to stimulate hMSCs’ exocytosis were not attributed to an increase of hMSCs.

### 2.2. Expression of CD9 Inhibited B16F10 Cell Migration in Wound Healing Assay

Before testing the impact of hMSC-derived exosomal CD9 in cancer cell migration, we transduced CD9 plasmids into melanoma B16F10 cells to identify the regulatory role of CD9 in cancer cell migration. As shown in Figure S2, B16F10 cells with ectopic CD9 expression (Figure S2A) showed a decreased wound healing activity (Figure S2B), which demonstrated that CD9 had an inhibitory effect on the migration ability of B16F10 cells in wound healing assay. Therefore, we wondered whether either dex-IO NP- or ionomycin-treated hMSC-derived exosomes could inhibit B16F10 cell migration via transferring their upregulated CD9 to B16F10 cells.

### 2.3. The Effect of hMSC-Conditioned Media in the Upper Chamber on B16F10 Cell Migration

**Figure 2:**
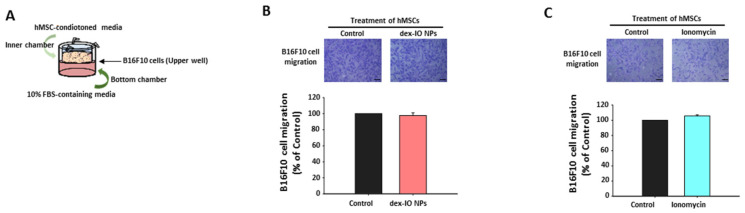
Effects of hMSC-conditioned media in the upper chamber on B16F10 cell migration in the transwell migration assay. (**A**) Schematic illustration of the experimental procedure. hMSCs were first treated without (Control) or with dex-IO NPs for 1 h or ionomycin for 30 min followed by wash, and then conditioned media from hMSCs incubated for 24 h were collected and added to the inner chambers. (**B**,**C**) Migration of B16F10 cells incubated with hMSC-conditioned media. Top panel: representative images of the migration of B16F10 cells by crystal violet staining. Bottom panel: quantification of the migration of B16F10 cells. Data are presented as the mean ± SEM of three independent experiments. Scale bar: 20 μm.

By adding hMSC-conditioned media containing exosomes (with or without treatment with dex-IO NPs or ionomycin) to the inner chambers (upper well with B16F10 cells) in the transwell migration assay, we examined B16F10 cell migration ability toward the bottom chamber, with 10% FBS-containing media as the attractant in the bottom chamber (Figure 2A). However, neither dex-IO NP-labeled hMSC-conditioned media (Figure 2B) nor ionomycin-incubated hMSC-conditioned media (Figure 2C), compared with control hMSC-derived media, had any impact on B16F10 cell migration. No significantly increased expression of CD9 was observed on dex-IO NP-labeled hMSC-conditioned media-treated B16F10 cells or ionomycin-incubated hMSC-conditioned media-treated B16F10 cells (data not shown); alternatively, because CD9 displayed opposing migration activities probably depending on associating molecules [17], it was speculated that an inadequate uptake of exosomal CD9 from dex-IO NP-labeled hMSC-conditioned media or ionomycin-incubated hMSC-conditioned media by B16F10 cells in the present study. Therefore, despite an inhibitory role of CD9 in B16F10 cell migration (Figure S2), these results seemed to disagree with the possibility that hMSCs could deliver their upregulated exosomal CD9 caused by dex-IO NPs or ionomycin to B16F10 cells to affect cell migration.

### 2.4. The Effect of hMSC-Conditioned Media in the Bottom Chamber on B16F10 Cell Migration

**Figure 3:**
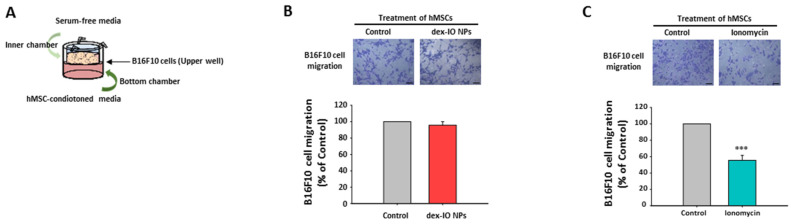
Effects of hMSC-conditioned media in the bottom chamber on B16F10 cell migration in the transwell migration assay. (**A**) Schematic illustration of the experimental procedure. hMSCs were first treated without (Control) or with dex-IO NPs for 1 h or ionomycin for 30 min followed by wash, and then conditioned media from hMSCs incubated for 24 h were collected and added to the bottom chambers. (**B**,**C**) Migration of B16F10 cells toward hMSC-conditioned media. Top panel: representative images of the migration of B16F10 cells by crystal violet staining. Bottom panel: quantification of the migration of B16F10 cells. Data are presented as the mean ± SEM of three independent experiments. *** *p* < 0.001 as compared with Control. Scale bar: 20 μm.

Surprisingly, when ionomycin-incubated hMSC-conditioned media (Figure 3C) but not dex-IO NP-labeled hMSC-conditioned media (Figure 3B) were added in the bottom chamber (Figure 3A), compared with control hMSC-derived media, B16F10 cells in the upper well showed a significantly reduced migration ability toward the bottom chamber. Given the fact that both dex-IO NPs and ionomycin could stimulate exosomal CD9 expression, it should be excluded that the involvement of exosomal CD9 in the reduced migration ability of B16F10 cells toward the bottom chamber was caused by ionomycin-incubated hMSC-conditioned media. Moreover, the data suggested that the reduced migration of B16F10 cells in the upper well toward the bottom chamber might be due to the decrease of migratory attraction of the ionomycin-incubated hMSC-conditioned media in the bottom chamber. In this context, we supposed that ionomycin but not dex-IO NPs attenuated the expression of specific chemokine(s) of hMSCs to result in reduced chemotactic migration of B16F10 cells.

### 2.5. Ionomycin-Incubated hMSC-Conditioned Media Reduced B16F10 Cell Migration by Chemokine CCL21 Decrease

**Figure 4:**
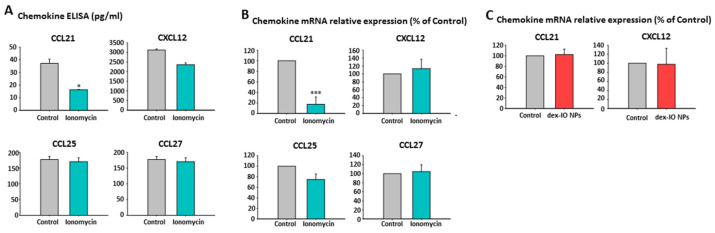
Effects of ionomycin and dex-IO NPs on hMSC’s chemokine production. (**A**) hMSCs were first treated without (Control) or with ionomycin for 30 min followed by wash, and then conditioned media from hMSCs incubated for 24 h were collected and processed for chemokine determination by ELISA. Data are presented as the mean ± SEM of three independent experiments. * *p* < 0.05 as compared with Control. hMSCs were first treated without (Control) or with ionomycin for 30 min (**B**) or dex-IO NPs for 1 h (**C**) followed by wash. After incubation with media for 24 h, hMSCs were incubated with media for 24 h and processed for cellular chemokine mRNA expression using RT-PCR. The relative mRNA levels are presented as the mean ± SEM of three independent experiments. *** *p* < 0.001 as compared with Control.

Concerned about the notable involvement of specific chemokine receptor expression on melanoma cells in their distinct metastatic dissemination [28], including CCR7, CCR9, CCR10, and CXCR4, we examined the contents of specific chemokines and found that CCL21 (corresponding to CCR7) but not CCL25 (corresponding to CCR9), CCL27 (corresponding to CCR10), and CXCL12 (corresponding to CXCR4) were markedly decreased in ionomycin-incubated hMSC-conditioned media compared with control hMSC-derived media by ELISA (Figure 4A). By real-time quantitative RT-PCR, we also demonstrated the marked decrease of CCL21 mRNA in ionomycin-incubated hMSCs (Figure 4B) but not in dex-IO NP-labeled hMSCs (Figure 4C), compared with control hMSCs. These results showed that ionomycin suppressed the expression and secretion of chemokine CCL21 in hMSCs, hence suggesting that the migration ability of B16F10 cells toward the bottom chamber was impeded by the lower chemo-attraction of decreased CCL21.

### 2.6. Silencing of hMSCs’ CCL21 Reduced B16F10 Cell Migration

**Figure 5:**
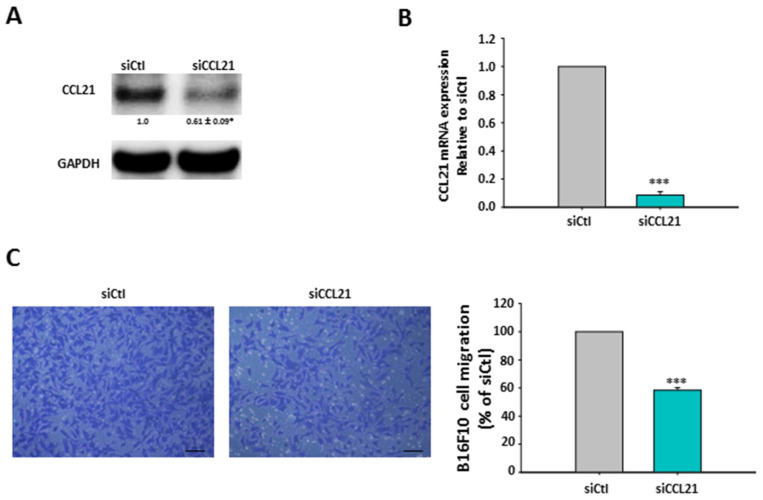
Effect of hMSCs’ CCL21 knockdown on B16F10 cell migration. Western blot analysis of intracellular CCL21 protein (**A**) and RT-PCR for intracellular CCL21 mRNA expression (**B**) in hMSCs infected with scrambled siRNA (siCtl) or CCL21 siRNA (siCCL21). Western blot images are shown as representative of at least three independent experiments with similar results and GAPDH was used as a loading control. The relative mRNA levels are presented as the mean ± SEM of three independent experiments. *** *p* < 0.001 as compared with siCtl. (**C**) Migration of B16F10 cells toward conditioned media from hMSCs infected with scrambled siRNA (siCtl) or CCL21 siRNA (siCCL21). Left panel: representative images of the migration of B16F10 cells by crystal violet staining. Scale bar: 20 μm. Right panel: quantification of the migration of B16F10 cells. Data are presented as the mean ± SEM of three independent experiments. *** *p* < 0.001 as compared with siCtl.

To directly examine the role of CCL21 in chemoattraction to B16F10 cells, CCL21 siRNA (siCCL21) was constructed to downregulate hMCSs’ CCL21 expression. siCCL21 indeed inhibited intracellular CCL21 protein expression and mRNA level as determined by western blot (Figure 5A) and RT-PCR (Figure 5B), respectively. By using transwell migration assay as in Figure 3A, a decreased migration ability of B16F10 toward the bottom chamber with media derived from hMSCs transfected with siCCL21 was observed than media derived from control hMSCs transfected with scrambled siRNA (siCtl) (Figure 5C). The results demonstrated that the CCL21 expression knockdown of hMSCs and subsequent decreased CCL21 secretion in media indeed exerted lower chemoattraction to B16F10 cells.

### 2.7. The Mechanism for the Opposite Effects of dex-IO NPs and Ionomycin on hMSCs’ CD9 Expression

**Figure 6:**
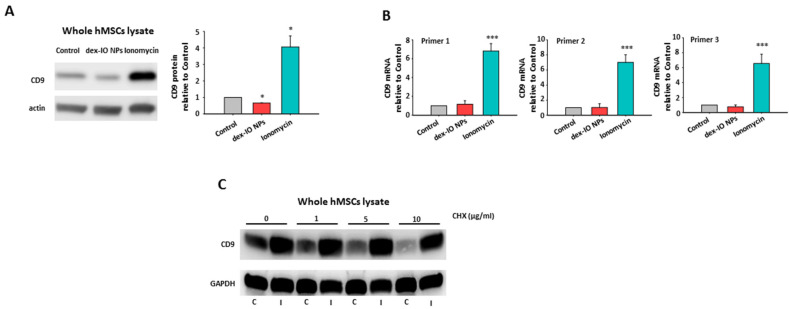
Ionomycin but not dex-IO NPs increased cellular CD9 expression via increasing CD9 transcript and protein stabilization. After treatment of hMSCs without (Control) or with dex-IO NPs for 1 h or ionomycin for 30 min, followed by wash, hMSCs were grown for 24 h and analyzed by Western blot for cellular CD9 protein (**A**) and RT-PCR for cellular CD9 mRNA (**B**). Western blot images are shown as representative of at least three independent experiments with similar results. * *p* < 0.05 as compared with Control. The relative CD9 mRNA levels are presented as the mean ± SEM of three independent experiments. *** *p* < 0.001 as compared with Control. (**C**) Cycloheximide (CHX) displayed a dose-dependent degradation of CD9 expression in hMSCs. Ionomycin antagonized CHX-dependent degradation of CD9. C: Control; I: ionomycin. GAPDH was used as a loading control. Data are representative of at least three independent experiments with similar results.

Although exosomal CD9 should not be involved in the reduced migration ability of B16F10 cells as shown in Figure 3, given that both dex-IO NPs and ionomycin could stimulate exosomal CD9 expression, we wondered about the effects of dex-IO NPs and ionomycin on cellular CD9 expression. Interestingly, as shown in Figure 6A, dex-IO NPs decreased but instead ionomycin increased cellular CD9 expression in hMSCs. By real-time quantitative RT-PCR, ionomycin but not dex-IO NPs was shown to be able to increase cellular CD9 transcript (Figure 6B). Moreover, cycloheximide (CHX), a protein synthesis inhibitor, displayed a dose-dependent degradation of CD9 expression; however, ionomycin efficiently prevented spontaneous and CHX-mediated CD9 degradation (Figure 6C), suggesting a stabilizing effect of ionomycin on CD9. The results indicated that ionomycin could increase cellular and exosomal CD9 expressions through increasing CD9 mRNA level as well as CD9 protein stabilization. On the other hand, because CD9 is constitutively expressed on exosomes, dex-IO NPs could only stimulate exocytosis to result in exosomal CD9 upregulation but not in cellular CD9 downregulation. The distinct effects of dex-IO NPs and ionomycin on cellular CD9 expression prompted us to explore the role of cellular CD9 upregulation in CCL21 decrease.

### 2.8. The Inhibitory Role of Ionomycin-Upregulated Cellular CD9 in hMSCs’ CCL21 Expression and B16F10 Cell Migration

**Figure 7:**
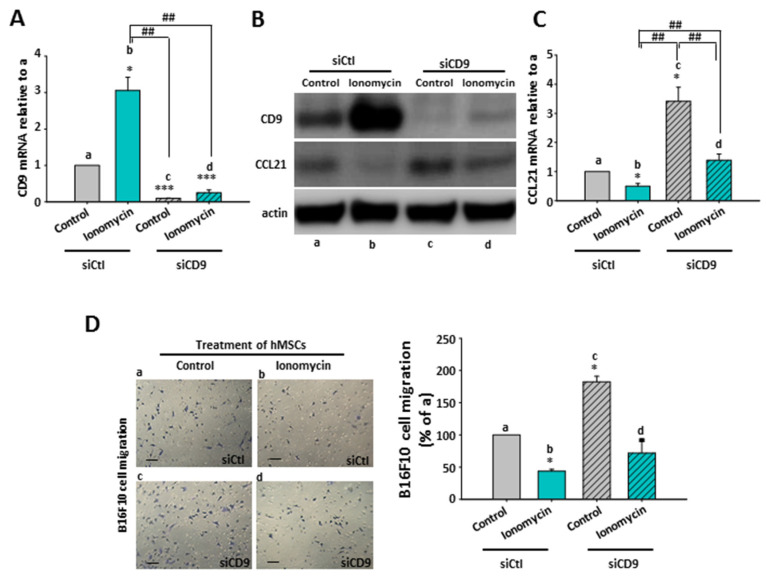
Effect of hMSCs’ CD9 knockdown on ionomycin-mediated hMSCs’ CCL21 downregulation and B16F10 cell migration inhibition. (**A**) CD9 siRNA (siCD9) downregulated hMSCs’ basal CD9 mRNA (**a** vs. **c**) and suppressed ionomycin-upregulated CD9 mRNA (**b** vs. **d**). The relative CD9 mRNA levels are presented as the mean ± SEM of three independent experiments. * *p* < 0.05; *** *p* < 0.001 as compared with (**a**). ## *p* < 0.01. (**B**) CD9 siRNA (siCD9) decreased cellular basal CD9 protein (**a** vs. **c**) and inhibited ionomycin-upregulated CD9 mRNA (**b** vs. **d**); however, CD9 siRNA (siCD9) increased cellular basal CCL21 protein (**a** vs **c**) but also reversed ionomycin-decreased cellular CCL21 protein (**b** vs. **d**) in hMSCs. Western blot images are shown as representative of at least three independent experiments with similar results, and actin was used as a loading control. (**C**) CD9 siRNA (siCD9) upregulated hMSCs’ basal CCL21 mRNA (**a** vs. **c**) but also inverted ionomycin-inhibited CCL21 mRNA (**b** vs. **d**). The relative CCL21 mRNA levels are presented as the mean ± SEM of three independent experiments. * *p* < 0.05 as compared with (**a**). ## *p* < 0.01. (**D**) B16F10 cells with ionomycin-incubated siCtl hMSC-derived conditioned media in the bottom chamber showed reduced cell migration (**a** vs. **b**). B16F10 cells with siCD9-infected hMSC-derived conditioned media in the bottom chamber showed increased cell migration (**a** vs. **c**). An antagonism between ionomycin and siCD9 in B16F10 cell migration was observed in the conditioned media from ionomycin-incubated hMSCs infected with siCD9 (**d**). Left panel: representative images of the migration of B16F10 cells by crystal violet staining. Scale bar: 20 μm. Right panel: quantification of the migration of B16F10 cells. Data are presented as the mean ± SEM of three independent experiments. * *p* < 0.05 as compared with (**a**).

Therefore, we further constructed specific siRNAs targeting CD9 to examine whether CD9 was involved in ionomycin-mediated CCL21 suppression, as shown in Figure 4. In hMSCs with control scrambled siRNA (siCtl), ionomycin was indeed able to induce cellular CD9 mRNA (Figure 7A, a vs. b) and protein (Figure 7B, a vs. b) expressions but decreased CCL21 protein expression (Figure 7B, a vs. b), like ionomycin did in control hMSCs (Figure 6). Transfection with CD9 siRNA (siCD9) not only reduced basal cellular CD9 mRNA (Figure 7A, a vs. c) and protein (Figure 7B, a vs. c) expressions but also markedly inhibited ionomycin-upregulated cellular CD9 mRNA (Figure 7A, b vs. d) and protein (Figure 7B, b vs. d). On the contrary, transfection with CD9 siRNA (siCD9) not only increased basal cellular CCL21 protein expression (Figure 7B, a vs. c) but also reversed ionomycin-decreased CCL21 protein expression (Figure 7B, b vs. d). Moreover, in control hMSCs with scrambled siRNA (siCtl), ionomycin was capable of decreasing CCL21 mRNA (Figure 7C, a vs. b); interestingly, silencing of CD9 (siCD9) could not only greatly increase basal cellular CCL21 mRNA (Figure 7C, a vs. c) expression but also dramatically reverse the level of ionomycin-decreased CCL21 mRNA (Figure 7C, b vs. d). These results demonstrated an inhibitory role of cellular CD9 in CCL21 expression in hMSCs.

### 2.9. The Effect of CD9-Regulated hMSCs’ CCL21 Expression and Secretion in B16F10 Cell Migration

Using the transwell migration assay as in Figure 3A, B16F10 cells in the upper well showed a significantly reduced migration ability toward the bottom chamber with ionomycin-incubated scrambled hMSC-conditioned media (Figure 7D, b) compared with control scrambled hMSC-derived media (Figure 7D, a); meanwhile, a lower CCL21 was expressed in ionomycin-incubated scrambled hMSC-conditioned media than control scrambled hMSC-derived media (Figure 7B,C, a vs. b). Accordingly, an increased migration ability toward the bottom chamber with media derived from hMSCs transfected with CD9 siRNA (siCD9) (Figure 7D, c) was observed in B16F10 cells in comparison to media derived from control hMSCs transfected with scrambled siRNA (siCtl) (Figure 7D, a). Certainly, CCL21 was increased in media derived from hMSCs transfected with CD9 siRNA (siCD9) (Figure 7B,C, c) in comparison to media derived from control hMSCs transfected with scrambled siRNA (siCtl) (Figure 7B,C, a). Similarly, a compromise was observed in B16F10 cell migration (Figure 7D, d) and CCL21 expression (Figure 7C, d) between ionomycin and CD9 siRNA. The data strongly suggested that ionomycin could upregulate cellular CD9 to decrease CCL21 expression and secretion of hMSCs, which reduced B16F10 cell migration due to chemoattraction reduction.

### 2.10. The Chemoattraction Reduction of Ionomycin-Incubated hMSC-Conditioned Media in Other Cancer Cell Lines

**Figure 8:**
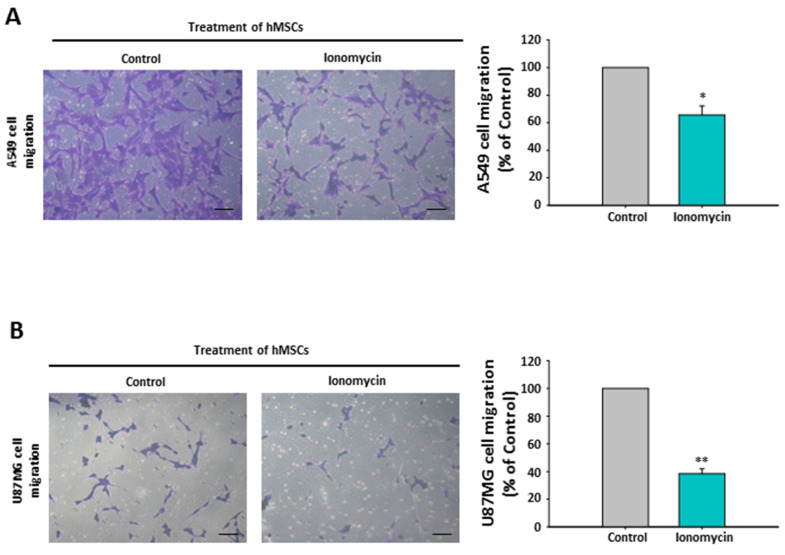
Effects of hMSC-conditioned media in the bottom chamber on A549 and U87MG cell migration in the transwell migration assay. Migration of A549 cells (**A**) and U87MG cell (**B**) toward hMSC-conditioned media. Left panel: representative images of the cell migration by crystal violet staining. Scale bar: 20 μm. Right panel: quantification of the cell migration. Data are presented as the mean ± SEM of three independent experiments. * *p* < 0.05 and ** *p* < 0.01 as compared with Control.

To further determine the significance of ionomycin-upregulated hMSCs’ CD9-mediated chemoattraction reduction (CCL21 decrease) in the model of cancer metastatic dissemination, the capacities of A549 and U87MG cells in the upper toward the bottom chamber were examined using transwell migration assay like in Figure 3A. Compared with control hMSC-derived media, ionomycin-incubated hMSC-conditioned media displayed reduced chemoattraction for A549 cells (Figure 8A) and U87MG cells (Figure 8B). Although the phenomenon of hMSCs’ CD9-regulated chemoattraction in a wide panel of cancer cells is worthy of additional investigation, these results demonstrate the important role of the CD9-CCL21 axis in cancer cell migration.

A large number of studies have focused on the role of cancerous tetraspanin CD9 expression in cancer migration and metastasis; however, this remains quite controversial. Exosomes derived from cancer cells are highly enriched with several tetraspanins, including CD9, which plays key functions of cancer exosomes between host cancer cells and recipient cells via endocytosis of exosomes to potentially affect the recipient cells [19,29,30]. On the contrary, cancer cells can be recipient cells for the uptake exosomal CD9 derived from non-cancerous cells in the tumor microenvironment, thereby inducing phenotypic and functional changes (e.g., cell migration) in these cancer cells, as described above [20]. These previous studies focus on the direct expression of CD9 in cancer cells. This is the first report about that CD9, expressed in non-cancerous cells, can indirectly regulate cancer cell migration.

**Figure 9:**
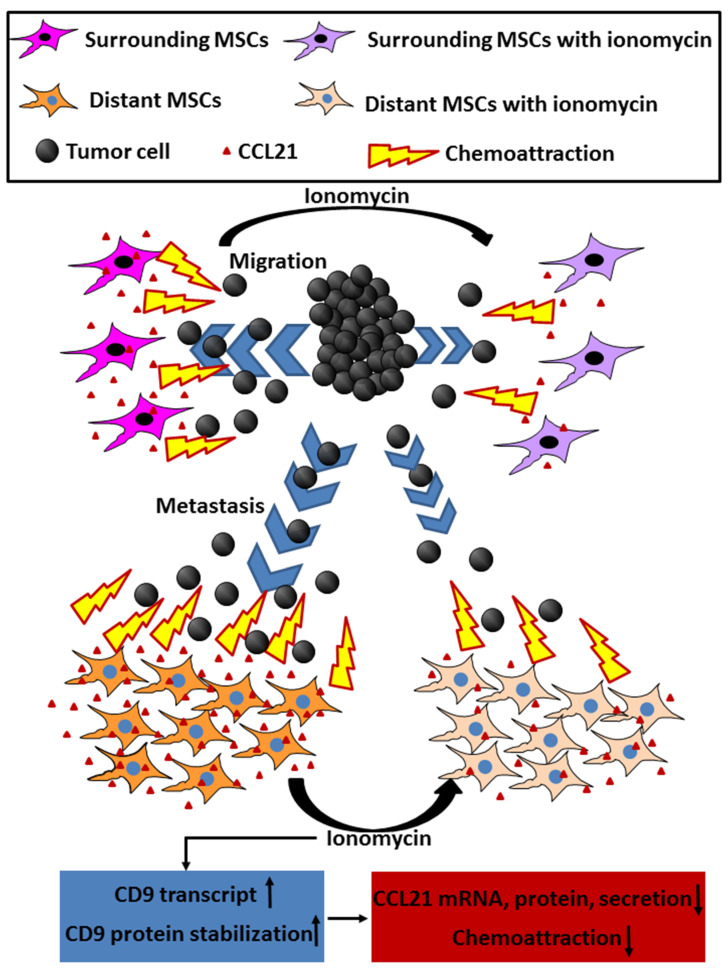
Schematic illustration of the role of the CD9-CCL21 axis pathway in regulating cancer cell migration/metastasis. CCL21 holds chemoattraction to cancer cells for migration and metastasis. Ionomycin is supposed to upregulate MSCs’ CD9 via increasing CD9 transcription as well as protein stabilization, to result in decreasing the CCL21 mRNA, protein and secretion, and the consequent lower chemoattraction.

Interestingly, by comparing ionomycin with dex-IO NPs, we excluded the possibility that hMSCs can be a donor cell of exosomal CD9 to affect cancer cell migration. Although ionomycin increased CD9 and cellular CD9 decreased CCL21 expression, the responsible mechanisms still need to be fully investigated. As illustrated in Figure 9, this study revealed a novel mechanism that intracellular CD9 upregulation in hMSCs can decrease CCL21 secretion to reduce cancer cell migration. Moreover, although the hMSCs in this study were derived from human bone marrow, it is highly acceptable to suspect whether other types of MSCs in the tumor microenvironment could affect cancer migration through CD9-CCL21 axis pathway. On the other hand, given the facts that bone marrow is one of the most common sites of cancer metastasis, and that CCL21 has been associated with the metastasis of various cancers [28], our findings highlight the important role that bone marrow-derived hMSCs’ CD9-mediated CCL21 regulation may have in bone metastasis. In addition, the CD9-CCL21 axis pathway regulating cancer cell migration suggests a new approach to developing new pharmaceutical strategies for prevention or/and therapy of cancer metastasis.

## 3. Materials and Methods

### 3.1. Cell Culture

Human mesenchymal stem cells (hMSCs) were isolated from bone marrow of normal donors using a Percoll density gradient centrifugation method, with informed consent approved according to the procedures of the Research Ethics Committee of the National Health Research Institutes (NHRI), Taiwan (EC1021003-E and EC1040506). hMSCs were cultured in regular growth medium consisting of low-glucose DMEM (Gibco) supplemented with 15% fetal bovine serum (FBS) (HyClone), 100 unit/mL of penicillin, and 100 μg/mL of streptomycin. Lung (A549) cancer cells were cultured in low-glucose DMEM supplemented with 10% FBS (Gibco), and glioma (U87MG) and melanoma (B16F10) cancer cells were cultured in high-glucose DMEM supplemented with 10% FBS (Gibco). All cells were maintained in a 5% CO2 atmosphere at 37 °C.

### 3.2. Preparation and Characterization of dex-IO NPs and Ionomycin Treatment

All chemicals are analytical grade without further purification. Iron (II) chloride tetrahydrate, Iron (III) chloride hexahydrate, and dextran (Mw ≈ 35,000–40,000) were purchased from Sigma Aldrich. The dextran-coated iron oxide nanoparticles were prepared by a chemical co-precipitation method as previously reported [23]. The data demonstrated the synthesis of dex-IO NPs (data not shown). The particle size of dex-IO NPs at 300 μg/mL in labeling medium was about 120 to 150 nm. The zeta potential of dex-IO NPs at 300 μg/mL in distilled water was about −5.5 mV. The particle size and surface charge were not significantly different with particle concentration or time (data not shown).

hMSCs were stimulated with 1 µM ionomycin (Merck KGaA, Darmstadt, Germany) for 30 min [27], or incubated with dex-IO NPs (300 μg/mL) or not in serum-free medium for 1 h at 37 °C [23]. Then hMSCs were washed once with PBS, then replaced with fresh serum-free media and incubated for one day at 37 °C in a humidified air atmosphere with 5% CO_2_.

### 3.3. Exosome Extraction and Identification

One day before preparation of the exosomes, hMSC culture medium was replaced with serum-free medium. Cell culture supernatants were collected after 24 h and centrifuged at 4000×*g* for 10 min to remove cells, dead cells, and debris. The exosomal fraction from different hMSC-conditioned media was isolated using ExoQuick-TC (EQ, System Biosciences Inc.; Mountain View, CA) according to the manufacturer’s recommendations. Media volumes were admixed with the EQ precipitation solution at 1:5 ratios and incubated overnight at 4 °C. At the end of the incubation time, the samples were centrifuged at 4000×*g* for 30 min, the supernatant was removed, and then the samples underwent a second round of centrifugation at 1500×*g* for 5 min. The pellet was processed for further analysis. The number of exosomes was determined by the NanoSight NS300 nanoparticle tracking analysis system (Malvern, UK).

### 3.4. Transwell Migration Assay

Studies on chemotactic migration of B16F10, A549, and U87MG cancer cells were performed using the Costar transwell chamber system (24-well; Costar) with membrane filters with a pore size of 8 μm. Samples, each containing 5 × 10^5^ cells in 200 μL of different hMSC-conditioned media, were added to the upper compartments (top chambers or inserts) and 600 μL of 10% FBS containing was placed in the lower chamber. Moreover, samples, each containing 5 × 10^5^ cells in 200 μL of serum-free media, were added to the upper compartments (top chambers or inserts) and 600 μL of different hMSC-conditioned media was placed in the lower chamber. After incubation overnight, cells on the top surface of the filters of the upper compartments were wiped off with cotton swabs. Cells that had migrated toward the lower surface of the filters were counted after staining with 0.5% crystal violet (Sigma). Triplicates of each sample were counted. Each migration experiment was performed in triplicate. The migration rate was expressed as the percentage variation of migrated cells with respect to the corresponding control as 100%.

### 3.5. Chemokine Protein Quantification

The changes in release of CCL21, CXCL12, CCL25, and CCL27 were evaluated using ELISA (enzyme-linked immunosorbent assay). The hMSCs (9.5 × 10^4^/well) were seeded into a 12-well plate and treated with 1μM ionomycin for 30 min. The cells were washed once by PBS, then replaced with fresh serum-free media and incubated for 24 h. The supernatants were collected and determined for CXCL12, CCL27 (PeproTech, Rocky Hill, NJ, USA), CCL21 (Thermo Fisher, Waltham, MA, USA) and CCL25 (Abcam, Cambridge, UK) using respective ELISA kits according to the manufacturers’ protocols. The absorbance was read at 450 nm using a SPECTRA max PLUS 384 (Molecular Devices, CA, USA) and data were analyzed with SoftMax Pro software (Molecular Devices, CA, USA).

### 3.6. CD9 and Chemokine mRNA Determination

Total RNA was extracted from hMSCs and converted into complementary DNA (cDNA) by a first-strand synthesis system (SuperScript III; Invitrogen). To determine the mRNA transcription level from cDNA, cDNA were analyzed by real-time PCR (ABI7900 Sequence Detector, Applied Biosystems) with the following thermal conditions: 95 °C for 10 min, and 40 cycles each for 95 °C for 15 s and 60 °C for 1 min. PCR was carried out using the following primers: CD9 primer 1, forward 5′- TGCAT CTGTA TCCAG CGCCA-3′ and reverse 5′- CTCAG GGATG TAAGC TGACT-3′; CD9 primer 2, forward 5′- GTGCA TGCTG GGACT GTTCT TCGGC TTC-3′ and reverse 5′- CACGC CCCCA GCCAA ACCAC AGCAG -3′; CD9 primer 3, forward 5′- TCTTG GTGAT ATTCG CCATT-3′ and reverse 5′- TTCGA GTACG TCCTT CTTGG-3′; CCL21, forward 5′- AACCA AGCTT AGGCT GCTCC ATCCC A-3′ and reverse 5′- TATGG CCCTT TAGGG GTCTG TGACC G-3′; CXCL12, forward 5′- TCAGC CTGAG CTACA GATGC-3′and reverse 5′- CTTTA GCTTC GGGTC AATGC-3′; CCL25, forward 5′- CCAAG GTGTC TTTGA GGACT GCTGC C-3′ and reverse 5′- GGGAG ACATT CCTCT TGCTG CTGCT G-3′; CCL27, forward 5′- TCCTG CTGCT GTCAT TGC-3′ and reverse 5′- GAGAG TGGCT TTCGG TAGAG -3′; GAPDH, forward 5′- GAGTC AACGG ATTTG GTCGT-3′ and reverse 5′- TTGAT TTTGG AGGGA TCTCG-3. Quantification was calculated using the ∆∆Ct method with GAPDH mRNA as endogenous control.

### 3.7. CD9 Silencing

The specific oligonucleotide sequences for human CD9 gene were as follows: 5′-GGAUUGCUGUCCUUGCCAUTT-3′ (sense) and 5′-AUGGCAAGGACAGCAAUCCTT-3′ (antisense). The siRNA control was 5′-AGGAGAUAUUUCGAGGCUUdTdT-3′ (sense) and 5′-AAGCCUCGAAAUAUCUCCUdTdT-3′ (antisense), which has no homology with relevant human genes. Transfection was carried out using TransIT-X2 (cat. no. MIR6003; Mirus, Madison, WI, USA) dynamic delivery system. Prior to transfection, cells (2.4 × 10^5^/well) were seeded into a 6-well plate and incubated in a humidified 5% CO_2_ atmosphere at 37 °C in antibiotic-free low-glucose DMEM medium supplement with 15% FBS for 24 h to about 80% confluence. For each transfection, 25 nM of either non-silencing siRNA control or specific siRNA were used; cells were transfected for 6 h in siRNA transfection medium with siRNA transfection reagent according to the manufacturer’s instructions. After transfection, fresh medium with antibiotics was added, and cells were grown for 24 h before further drug treatment.

### 3.8. Western Blot Analysis

hMSCs (6 × 10^5^ cells) were incubated with regular cultured medium in 100 mm dishes. The hMSCs were treated with 1 μM ionomycin for 30 min or dex-IO NPs (300 μg/mL) in serum-free medium for 1 h at 37 °C. After treatment cells were rinsed with ice-cold 1×PBS and were lysed by the addition of lysis buffer (25 mM HEPES, pH 7.5, 150 mM NaCl, 1% Igepal CA-630, 10 mM MgCl_2_, 1 mM EDTA, 2% glycerol, 1 μM phenylmethylsulfonyl fluoride, 1 μg/mL leupeptin, and 10 μg/mL aprotinin) for 1 h at 4 °C. The suspensions were centrifuged at 13,000 rpm for 30 min at 4 °C. The protein concentration of the supernatant was assessed by the Bio-Rad protein assay kit. Proteins were separated by electrophoresis in a gradient polyacrylamide gel and transferred to a polyvinylidene difluoride membrane. Then the membranes were incubated at room temperature in 0.1% Tween 20 with TBS plus 5% bovine serum albumin (BSA) for 1 h. The membranes were incubated with rabbit anti-CD9 antibodies (cs13174; dilution 1:1000, Cell Signaling Technology, Topsfield, MA, USA), rabbit anti-CCL21 antibodies (A1896; dilution 1:1000, ABclonal Technology, Woburn, MA, USA), and mouse anti-actin antibodies (sc47778; dilution 1:1000, Santa Cruz Biotechnology, Santa Cruz, CA, USA) in TBST containing 5% BSA at 4 °C, and then washed three times in TBST, for 10 min each time. After washing, HRP-conjugated anti-rabbit (cs7074; dilution 1:5000, Cell Signaling Technology) or anti-mouse (cs7076, dilution 1:5000, Cell Signaling Technology) antibodies were incubated with membranes for 1 h at room temperature. After washing, the membranes were developed using the Immobilon Western chemiluminescent HRP substrate kit (Millipore, Burlington, MA, USA).

### 3.9. Statistical Analysis

Data are presented as the mean (standard error of the mean) for the indicated numbers of separate experiments. The results were compared using Student’s-test in the case of two groups. Statistical significance was assigned if the probability value (*p*) was less than 0.05.

## Data Availability

Not applicable.

## Supplementary Materials

The following are available online at https://www.mdpi.com/1422-0067/22/4/1738/s1. Figure S1: Effects of dex-IO NPs and ionomycin on the cell viability of hMSCs and Figure S2: Effect of CD9 overexpression in B16F10 cell migration.

## Abbreviations

hMSCs/MSCs	(Human) Mesenchymal stem cells
EVs	Extracellular vesicles
miRNAs/miR	Micro RNAs
CAF	Cancer-associated fibroblstas
IO	Iron oxide
NPs	Nanoparticles
EVs	Extracellular vesicles
miRNAs/miR	Micro RNAs
CAF	Cancer-associated fibroblstas
IO	Iron oxide
NPs	Nanoparticles
MRI	Magnetic resonance imaging
EGF/EGFR	Epidermal growth factor (receptor)
CCL	C-C motif chemokine ligand
CCR	C-C motif chemokine receptor
CXCL	C-X-C motif chemokine ligand
CXCR	C-X-C motif chemokine receptor
mRNA	Messenger RNA
CHX	Cycloheximide
siRNA	Small interfering RNA
