# Supplementary material for: CD9 Upregulation-Decreased CCL21 Secretion in Mesenchymal Stem Cells Reduces Cancer Cell Migration
Source: Int J Mol Sci. 2021 Feb 9;22(4):1738. doi: 10.3390/ijms22041738 (PMC7915477; doi:10.3390/ijms22041738)
Supplement: Supplementary file 1 [file ijms-22-01738-s001.pdf]

## Supplementary Data

# CD9 Upregulation-Decreased CCL21 Secretion in Mesenchymal Stem Cells Reduces Cancer Cell Migration

*In Vitro Cytotoxicity of dex-IO NPs and Ionomycin on hMSCs*

*Cell Viability Assay*

The cytotoxic effects of dex-IO NPs and ionomycin on hMSCs were performed using MTT assays. hMSCs were seeded onto 96-well plates at a density of 8 × $10^3$ cells/well, after overnight incubation cells were treated without (Control) or with 300 ug/ml dex-IO NPs [1] for 1 h or 1 uM ionomycin [2] for 30 min follwed by wash, and then allowed to grow for 24 h. Then MTT reagent (Sigma-Aldrich, St. Louis, MO, USA) was added to the medium in each well and incubated for 4 h at 37 °C. The supernatant was removed carefully. The formazan crystals were then dissolved in 100 µl of dimethyl sulfoxide (DMSO, Sigma-Aldrich, St. Louis, MO, USA). Cell viability in each well was determined by optical density measurement at 570 nm on micro plate reader.

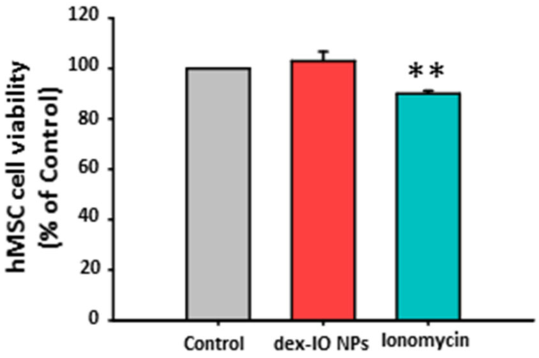

**Figure S1.** Effects of dex-IO NPs and ionomycin on the cell viability of hMSCs. hMSCs were treated without (Control) or with dex-IO NPs for 1 h or ionomycin for 30 min followed by wash, and then allowed to grow for 24 h. The cell viability was assessed by MTT assay. Ionomycin caused a significant but slight decrease of cell viability. Data are presented as the mean ± SEM of three independent experiments. $^{**}p < 0.01$ as compared with Control.

*Ectopic Expression of CD9 Inhibited B16F10 Cell Migration in Wound Healing Assay*

*Ectopic Expression of CD9 and Wound Healing Assay*

For transfecting experiments, B16F10 ($3.6 \times 10^5$ cells/well) were seeded on a six-well culture plate and then were transfected with CD9-flag plasmid (Sino Biological Inc., USA) or empty vector (pcDNA3.1-flag) using Maestrofectin Transfection Reagent (Omicsbio, Taipei City, Taiwan) according to the manufacturer's instructions. Ectopic expression of CD9 was examined by Western Blot. After 24 h incubation in complete media, cells were scratched using a sterile 200 μl pipette tip, and incubated another 24 h. The scratch area was monitored under phase-contrast microscope at 0 and 24 h. Scratch width was measured in 5 randomly selected areas at 10x magnification using ImageJ software.

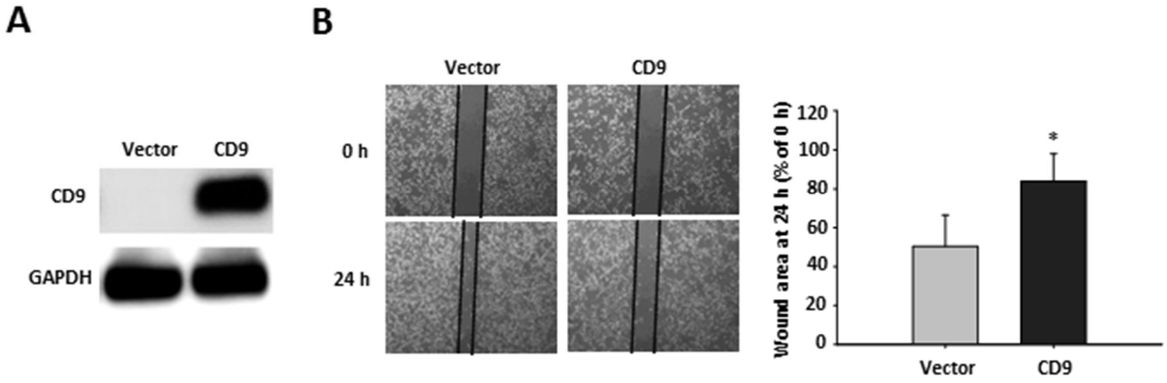


**Figure S2.** Effect of CD9 overexpression in B16F10 cell migration. (**A**) Western blot analysis of CD9 in the whole cell lysate of B16F10 cells infected with control vector or CD9 construct. GAPDH was used as a loading control. Shown are representative of at least three independent experiments with similar results. (**B**) Scratch-wound assay showing the migration of the B16F10 cells infected with control vector or CD9 construct (left panel). Quantification of the migration of the B16F10 cells infected with control vector or CD9 construct (right panel) as determined by wound area. Data are presented as the mean ± SEM of three independent experiments. $^{*}p < 0.05$ as compared with vector.

References

1. Chung, T.-H.; Hsieh, C.-C.; Hsiao, J.-K.; Yao, M.; Hsu, S.-C.; Huang, D.-M. Dextran-coated iron oxide nanoparticles turn protumor mesenchymal stem cells (MSCs) into antitumor MSCs. RSC Adv. **2016**, *6*, 45553–45561.

2. Kumar, J.D.; Holmberg, C.; Balabanova, S.; Borysova, L.; Burdyga, T.; Beynon, R.; Dockray, G.J.; Varro, A. Mesenchymal stem cells exhibit regulated exocytosis in response to chemerin and IGF. PLoS ONE **2015**, *10*, e0141331.
